# One-shot vaccination with an insect cell-derived low-dose influenza A H7 virus-like particle preparation protects mice against H7N9 challenge^☆^

**DOI:** 10.1016/j.vaccine.2013.11.036

**Published:** 2014-01-09

**Authors:** Miriam Klausberger, Monika Wilde, Dieter Palmberger, Rong Hai, Randy A. Albrecht, Irina Margine, Ariana Hirsh, Adolfo García-Sastre, Reingard Grabherr, Florian Krammer

**Affiliations:** a Vienna Institute of BioTechnology, University of Natural Resources and Life Sciences, Vienna, Austria; b Department of Microbiology, Icahn School of Medicine at Mount Sinai, New York, NY, USA; c Global Health and Emerging Pathogens Institute, Icahn School of Medicine at Mount Sinai, New York, NY, USA; d Graduate School of Biological Sciences, Icahn School of Medicine at Mount Sinai, New York, NY, USA; e Department of Medicine, Division of Infectious Diseases, Icahn School of Medicine at Mount Sinai, New York, NY, USA

**Keywords:** Influenza, H7N9, Pandemic, Virus-like particle, Baculovirus, Cross-reactivity

## Abstract

Human infections with a novel influenza A H7N9 subtype virus were reported in China recently. The virus caused severe disease with high mortality rates and it raised concerns over its pandemic potential. Here, we assessed in the mouse model protective efficacy of single immunisations with low vaccine doses of insect cell-derived H7 virus-like particles, consisting of hemagglutinin and matrix protein. Vaccinated mice were fully protected and survived a stringent lethal challenge (100 mLD50) with H7N9, even after a single, unadjuvanted, low vaccine dose (0.03 μg). Serum analysis revealed broad reactivity and hemagglutination inhibition activity across a panel of divergent H7 strains. Moreover, we detected significant levels of cross-reactivity to related group 2 hemagglutinins. These data demonstrate that virus-like particle vaccines have the potential to induce broadly protective immunity against the novel H7N9 virus and a variety of other H7 strains.

## Highlights

• Creation of influenza H7-VLPs containing HAs from A/Shanghai/1/13 or A/Anhui/1/13.
• Single low-dose vaccination is 100% protective against stringent SH1 challenge.
• Single immunisation induces HI titres of up to 1:40.
• Induction of broadly sero-reactive and HI-active antibodies.
• Baculovirus impurities might beneficially contribute to protective efficacy.

## 1 Introduction

On March 31, 2013 the Chinese public health authorities reported three cases of laboratory-confirmed human infection with a novel avian-origin influenza A H7N9 virus [1]. Two patients in Shanghai and one in the surrounding Anhui province were hospitalised with symptoms of cough, dyspnoea and high fever and developed acute respiratory distress syndrome (ARDS) and pneumonia complications, which proved to be deadly [2]. As of October 25, 2013 [3], 137 human cases of influenza A H7N9 infection were reported to the WHO, including 45 deaths. This is the highest mortality number attributed to H7 infections worldwide to date. Efforts to restrict avian to human transmission were initiated including shutting down large poultry markets throughout the country. Antivirals are currently the only prophylactic and therapeutic options available for human use. Since the novel H7N9 strains are resistant to M2 ion channel blockers [2], [4], neuraminidase inhibitors are recommended as frontline therapeutic by the Chinese Center for Disease Control and Prevention [5]. However, oseltamivir-resistant viruses have been associated with antiviral treatment and poor clinical outcome [6], [7]. The exceptional adaptive ability of the virus and the lack of human pre-immunity and of available vaccines underline the necessity of rapid measures to be taken and research on the development on human H7 vaccines is underway [8], [9], [10], [11], [12], [13], [14].

Here, we assess the efficacy of a single low vaccine dose of influenza A H7 virus-like particles (VLPs) of Avian Influenza A (H7N9) virus origin to protect against a stringent viral challenge in the mouse model. Two-component influenza virus-like particles, containing HAs from the first H7N9 virus isolates (A/Anhui/1/13 or A/Shanghai/1/13, respectively) and the matrix protein (M1) from A/Udorn/307/1972, were produced in the *Trichoplusia ni* insect cell line High Five (BTI-TN-5B1-4) using the baculovirus expression system. Previous studies conclusively demonstrated the potent immune stimulating properties of live baculovirus in vaccine preparations [15], [16]. Hence, in order to keep the by-product in the vaccine formulation, we concentrated the VLPs and residual baculovirus from the culture supernatant by one-step sucrose-cushion purification. Mice received one VLP vaccine dose containing different amounts of HA (3 μg, 0.3 μg and 0.03 μg) and 5 weeks later were challenged with a stringent viral dose (100 mLD50) of the A/Shanghai/1/13 H7N9 strain. Pre-challenge serum was evaluated for the breadth of reactivity and hemagglutination inhibition (HI) activity of the elicited humoral response to divergent H7 HAs, as well as representatives of all group 2 HA subtypes.

Even the lowest tested vaccine doses conferred full protection against the stringent viral challenge. In addition, a single vaccination with the H7 VLP vaccine induced serum antibodies that were broadly reactive and HI active against divergent H7 subtyped viruses. We also detected sero-reactivity to heterosubtypic members of the group 2 HAs, such as H15 and H3.

## 2 Materials and methods

### 2.1 Insect cells

Sf9 insect cells (ATCC # CRL-1711) were routinely propagated at 27 °C in TNM-FH medium (Gemini Bio-Products, West Sacramento, CA) supplemented with 0.1% (v/v) Pluronic 68 (Sigma, St. Louis, MO), 10% (v/v) foetal bovine serum (FBS) (Atlanta Biologicals, Norcross, GA) and Penicillin–Streptomycin antibiotic mixture (Life Technologies, Carlsbad, CA). For baculovirus amplification, the medium was switched to 3% (v/v) FBS. BTI-TN-5B1-4 (High Five – Vienna Institute of Biotechnology subclone) [17] cells were used for expression of VLPs and maintained at 27 °C in custom modified serum-free IPL-41 medium (PAN-Biotech GmbH, Aidenbach, Germany) at 27 °C as described in [18] supplemented with Penicillin–Streptomycin antibiotic mixture.

### 2.2 Viruses

Recombinant influenza viruses were generated by reverse genetics as described before [19], [20], [21]. Recombinant A/Puerto Rico/8/34 (PR8) viruses (6:2) were generated that expressed HA and NA of human H7N9 viruses from the current outbreak in China, including A/Shanghai/1/13 (abbreviated PR8:SH1) and A/Anhui/1/13 (PR8:AH1). The nucleotide sequences of the HA and NA of SH1 and AH1 were downloaded from the GISAID Epiflu database (accession numbers EPI439486 and EPI439507, respectively). Gene synthesis was conducted by GeneArt (Life Technologies, Carlsbad, CA). SH1 and AH1 HA and NA sequences were subcloned into the ambisense rescue plasmid pDZ for rescue of recombinant influenza viruses. Additional recombinant PR8 virus (7:1) were generated that expressed the HA of the H7 Eurasian lineage virus A/mallard/NL/12/00 (H7N3; PR8:malNL00), or the HA of A/chicken/Jalisco/12283/12 (H7N3; PR8:chickJal12) which was genetically modified to remove the multibasic cleavage site. An additional recombinant PR8 viruses was included that expressed a chimeric cH7/3 HA in which the globular head domain was derived from the H7 North American lineage virus A/mallard/Alberta/24/01 (H7N3; PR8:malAlb01) on an H3 stalk [21], [22]. Viruses were propagated in 8- to 10-day-old specific pathogen-free embryonated chicken eggs (Charles River Laboratories) for 48 h at 37 °C and virus was titred on MDCK cells in the presence of tosyl phenylalanyl chloromethyl ketone (TPCK) treated trypsin.

### 2.3 Production of virus-like particles in insect cells

Synthesised SH1 and AH1 HA genes (GISAID Epiflu database accession numbers EPI439486 and EPI439507, respectively) and the matrix protein (M1) gene from strain A/Udorn/307/72 (H3N2) (GenBank: DQ508932.1), synthesised by Sloning (Puchheim, Germany), were cloned as previously described [17]. VLPs consisting of the respective H7 HA (either AH1 or SH1) and the matrix protein (M1) from the unrelated H3N2 subtype were produced by baculovirus infection of insect cells as described before [17]. Empty VLPs consisting of M1 only were prepared to be used as a negative control.

Briefly, the synthetic genes were cloned into a modified pFastBacDual baculovirus transfer vector and recombinant bacmids were constructed using the Bac-to-Bac System (Invitrogen, Carlsbad, CA). Recombinant baculovirus was rescued from Sf9 cells and amplified. VLPs were expressed in High Five cells using Fernbach flasks incubated at 27 °C. Cells were infected with the recombinant baculoviruses at a multiplicity of infection of approximately 5 and culture supernatant was harvested 4 days post infection by low-speed centrifugation (3.000 rpm, 10 min). VLPs were partially purified and concentrated using a 30% (w/v) sucrose cushion in phosphate buffered saline (PBS) and the pellet was resuspended in PBS and stored at 4 °C.

### 2.4 Western blot analysis and HA quantification

To quantify the HA content of the VLPs, different concentrations of VLP samples were compared to known concentrations of recombinant His-tag purified SH1-HA containing a T4 foldon trimerisation domain [23]. VLP and His-tag HA were separated by SDS-PAGE using 4–12% gradient polyacrylamide gels (Invitrogen, Carlsbad, CA). After electrophoretic transfer onto polyvinylidene difluoride (PVDF) membranes (Thermo Fisher Scientific, Waltham, MA), the membranes were blocked for 1 h in PBS containing 0.1% Tween 20 (v/v) (PBST) and 3% (w/v) non-fat dry milk powder. After three washes with PBST, the blots were incubated for 3 h with convalescent serum obtained from mice sublethally infected with SH1 at a dilution of 1:1000. Membranes were washed three times with PBST and incubated for 1 h at room temperature with a horseradish-peroxidase-conjugated goat anti-mouse IgG (H + L) secondary antibody (Santa Cruz Biotechnology, Inc., Dallas, TX) at a dilution of 1:2000. Then, membranes were rinsed again and protein bands were visualised using the two-component Western Lightning^®^ Plus-ECL enhanced chemiluminescence substrate kit (PerkinElmer, Inc., Waltham, MA) and Ultra Cruz™ Autoradiography Blue Films (Santa Cruz Biotechnology, Inc., Dallas, TX). Radiographs were developed on a SRX-101A processor (Konica Minolta, Osaka, Japan). HA content of the VLP samples was determined densitometrically against known concentrations of the SH1-HA protein using ImageJ (National Institutes of Health).

### 2.5 Hemagglutination assay

Two-fold serial dilutions of PR8:AH1, PR8:SH1, PR8:malNL00, PR8:malAlb01 and PR8:chickJal12 recombinant reassortant virus strains in PBS (50 μL) were prepared in Nunc^®^ 96-well polystyrene V-bottom microwell plates (Thermo Fisher Scientific, Waltham, MA), followed by the addition of 50 μL 0.5% (v/v) chicken or turkey red blood cells (RBCs) (Lampire Biological Laboratory, Pipersville, PA) in PBS into each well. RBCs were allowed to settle for 45–60 min at 4 °C and the HA titre was determined by visual inspection. Hemagglutination units (HAU) are read as the reciprocal of the last dilution, giving rise to hemagglutination of red blood cells.

### 2.6 Quantification of baculovirus content in the vaccine preparation

Baculovirus titres in the VLP vaccine doses were determined by plaque assay on Sf9 cells with minor modifications as described in [24]. Briefly, the assay was carried out in 6-well plates in duplicates. After seeding 1 × 10^6^ cells per well, the cells were allowed to attach to the surface, medium was removed and 200 μL of the diluted VLP vaccine formulations (10-fold dilutions in TNM-FH unsupplemented) were added and incubated for 1 h at 27 °C with periodic shaking. After infection, the samples were removed and cells were overlaid with 2 mL of a solution containing 1% agarose in TNM-FH, 10% (v/v) foetal bovine serum, Penicillin–Streptomycin antibiotic mixture pre-warmed to 37 °C. The plates were incubated at 27 °C for 6 days and plaques were counted after live-cell staining with 200 μL of 5 mg/mL Thiazolyl blue formazan MTT (Sigma, St. Louis, MO) for 3–4 h.

### 2.7 Vaccine formulation

SH1-VLPs were prepared in three different concentrations in PBS as per HA content (3 μg, 0.3 μg and 0.03 μg SH1-HA per 50 μL vaccine dose). The AH1-VLP vaccine was prepared at a single concentration (0.3 μg AH1-HA per 50 μL). M1-VLPs served as a negative control and were adjusted to a total protein concentration equal to that of SH1-VLP (0.3 μg HA content) as measured using the Quickstart Bradford Dye Reagent (Bio-Rad, Hercules, CA) with bovine serum albumin as standard. The content of infectious baculovirus per μg HA varied slightly between 1.03 × 10^7^ and 2.62 × 10^7^ pfu (plaque forming units). All vaccine doses including the M1-only VLP negative control contained similar doses of infectious baculovirus (Suppl. Table 1). For further characterisation the migration pattern of the VLPs into a sucrose gradient was analysed by ultracentrifugation (Suppl. Fig. 1).

### 2.8 Mouse immunisation and challenge

Animal experiments were performed using 6–8 week-old female BALB/c mice (Jackson Laboratories) according to the guidelines of the Icahn School of Medicine at Mount Sinai Institutional Animal Care and Use Committee (permit LA12-00028). Animals had free access to food and water and were kept on a 12-h light/dark cycle. Mice were anesthetised by intraperitoneal (IP) injection of 0.1 mL of a ketamine/xylazine mixture (0.15 mg/kg and 0.03 mg/kg) before intranasal procedures. The prime-only group was immunised once with SH1-VLPs at a dose of 0.03 μg, 0.3 μg or 3 μg based on HA content in PBS or with 0.3 μg AH1-VLPs in a volume of 50 μL intramuscularly (i.m.) in the calf muscle (*N* = 5 per vaccine dose) at day 0. The prime-boost group (*N* = 5) was immunised twice with 0.3 μg SH1-VLPs, at an interval of 14 days. A control group (*N* = 5) was immunised once with M1-only VLPs at a total protein concentration equal to that of the SH1-0.3 μg vaccine dose. CD8^+^-depleted prime-only groups received one immunisation with 0.3 μg SH1- or M1-VLPs (*N* = 5) and were treated by IP injection of 300 μg of anti-CD8^+^ T-cell antibody [25] (from hybridoma line 2.43) for CD8^+^ T-cell depletion 48 and 24 h prior to challenge. Naive mice (*N* = 5 per group) were included as additional negative control group. Three weeks after the last immunisation blood was drawn from anesthetised mice by submandibular bleeding. Mice were then infected with 100 LD_50_ of the recombinant virus PR8:SH1. Weight loss was monitored daily for up to 14 days and animals that lost 25% or more of their initial body weight were scored dead and humanely euthanised, according to institutional guidelines.

### 2.9 ELISA assays

A quantitative ELISA was performed to assess titres of HA-specific IgG. Sera (*N* = 5) from the different vaccine groups were pooled and assayed in duplicate. HA proteins of representatives of all influenza A group 2 subtypes were recombinantly expressed with a C-terminal T4 foldon trimerisation domain and an N-terminal His-tag as described in [23] and used as antigens (HAs from A/Shanghai/1/13 (H7N9, abbreviated SH1), A/Anhui/1/13 (H7N9, AH1), A/mallard/NL/12/00 (H7N3, malNL00), A/rhea/North Carolina/39482/93 (H7N1, rheaNC93), A/chicken/Jalisco/12283/12 (H7N3, chickJal12), A/Hong Kong/1/68 (H3N2, H3), A/duck/Czech/56 (H4N6, H4), A/mallard/Interior Alaska/10BM01929/10 (H10N7, H10), A/mallard/Gurjev/263/82 (H14N5, H14), A/wedge tailed shearwater/Western Australia/2576/79 (H15N9, H15) and A/California/04/09 (pandemic H1N1, pH1)).

Briefly, Immulon 4HBX^®^ ultra-high-binding polystyrene plates (Thermo Fisher Scientific, Waltham, MA) were coated with rHA at a concentration of 2 μg/mL in coating buffer (0.1 M Na_2_CO_3_/NaHCO_3_, pH 9.2, 50 μL/well) at 4 °C overnight. Plates were blocked with PBST and 3% (w/v) non-fat dry milk for 2 h at room temperature. Plates were incubated with 3-fold dilutions to endpoint titre of pooled serum samples (100 μL per well in PBST with 1% non-fat dry milk (starting concentration: 1:50 dilution) for 2 h at room temperature. Following three washes with 100 μL PBST, plates were incubated with horseradish-peroxidase-conjugated anti-mouse IgG (Santa Cruz Biotechnology, Dallas, TX) at a dilution of 1:3000 in PBST and non-fat dry milk (1%, v/v) for 1 h at room temperature. Unbound antibody was removed by three washes with 100 μL PBST and plates were developed using SigmaFAST OPD substrate (Sigma, St. Louis, MO) (100 μL/well) and stopped with 3 M HCl (50 μL/well). The colorimetric change was measured as the optical density (OD 490 nm) on a Synergy 4 (BioTek, Winooski, VT) microplate reader. The endpoint titre was defined as the reciprocal of the highest dilution that yields an OD-value above the mean plus three standard deviations of blank wells.

### 2.10 Hemagglutination inhibition assay

The hemagglutination inhibition (HI) assay was used to assess functional antibodies to the HA able to inhibit agglutination of turkey red blood cells (tRBCs). Serum samples were treated with 4 volumes of a receptor-destroying enzyme of *Vibrio cholera* filtrate (Sigma, St. Louis, MO) for 18 h at 37 °C. After addition of 3 volumes of 2.5% (v/v) sodium citrate, the serum samples were incubated at 56 °C for 30 min and diluted with PBS to yield a 1:10 dilution of the original serum sample. Serum samples were 2-fold serially diluted in PBS (25 μL sample volume) in Nunc^®^ 96-well polystyrene V-bottom microwell plates (Thermo Fisher Scientific, Waltham, MA) and then incubated with recombinant reassortant virus (PR8:AH1, PR8:SH1, PR8:malNL00, PR8:malAlb01 or PR8:chickJal12) at 4 HAU/25 μL in PBS for 30 min at room temperature. Then, 50 μL 0.5% tRBCs (Lampire Biological Laboratory, Pipersville, PA) were added and the mixture was incubated for 45 min at 4 °C. Sera from all groups were assayed individually for the challenge strain PR8:SH1 for HI activity. Divergent H7 strains were assayed with pooled sera. The HI titre was calculated from the reciprocal of the highest dilution that completely inhibited hemagglutination of red blood cells and the geometric mean titre (GMT) of two independent assays was reported as the final titre. Two negative HI readings were assigned <10, single negative results were scored a value of 5 for the calculation of geometric means.

## 3 Results

### 3.1 Efficacy of H7-VLPs in an *in vivo* mouse challenge experiment

In preparedness for a potential H7N9 pandemic, it is highly desirable, not only for vaccine manufacturers, but also for health care providers, to develop an influenza vaccine that at low vaccine dose, most preferably with a single administration, stimulates good immune responses. In a previous study by Margine and colleagues [16] showed that residual baculovirus in VLP formulations enhance the immunogenicity of the vaccine due to potent stimulation of the innate immune response and by biasing IgG isotype distribution to the more active IgG2 subtype.

**Fig. 1:**
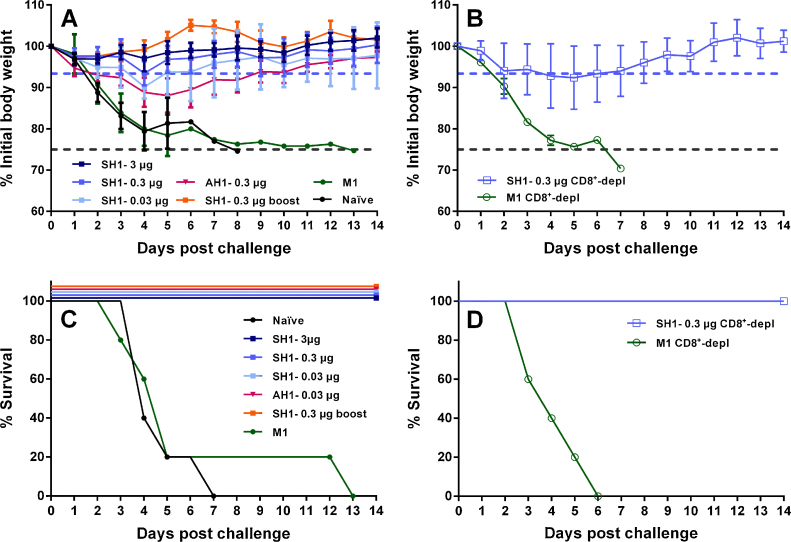
VLP vaccines protect against lethal H7N9 challenge in mice. Mice (*N* = 5 per group) were intramuscularly vaccinated with the following vaccine preparations and were intranasally challenged with 100 mLD50 of A/Shanghai/1/13. Weight loss (A and B) and survival rates (C and D) were monitored for 14 days post challenge. BALB/c mice received one dose of the vaccine preparation intramuscularly on day 0 of SH1-VLPs with a HA content of 3 μg (dark blue), 0.3 μg (signal blue), 0.03 μg (light blue), AH1-VLPs containing 0.3 μg HA (pink), M1-VLPs at a total protein concentration equal to that of SH1-0.3 μg VLPs (dark green) or were not vaccinated (black). Another group of mice received two vaccinations at days 0 and 14 with 0.3 μg SH1-VLPs (orange). Control groups were either vaccinated with 0.3 μg SH1-VLPs (signal blue, open squares) or M1-VLPs (dark green, open circles) and were CD8^+^ T-cell depleted by antibody treatment 48 h and 24 h pre challenge and weight loss (B) and survival (D) were followed for 14 days. The weight loss curves represent the mean percentage of the group initial body weight and error bars indicate the standard deviation. Grey baselines (A and B) indicate the weight cut-off of 75% of the initial body weight at which mice were humanly euthanised. The signal blue baselines in (A and B) indicate the maximum weight loss of undepleted SH1-0.3 μg-vaccinated mice to allow for comparison of weight loss kinetics with the CD8^+^ T-cell depleted mice that received the same vaccine dose.

Thus, we evaluated whether unadjuvanted single immunisations with low doses of our VLP-vaccine containing baculovirus were effective in eliciting protective immune responses in an *in vivo* mouse experiment using a stringent 100 mLD50 challenge dose. We assessed protection conferred by three different concentrations of SH1-VLPs (3 μg, 0.3 μg and 0.03 μg in terms of HA content, administered intramuscularly). We also compared groups that received a single vaccine dose with a group that received two immunisations on days 0 and 14 (0.3 μg in terms of HA content). To explore whether a prime-only strategy could protect against a heterologous strain as well, we included a VLP formulation that contained HA of AH1, a divergent H7N9 isolate. [4]. Mice that received two immunisations with 0.3 μg SH1, expectedly showed a 100% survival rate and little weight loss (Fig. 1A and C). Similarly, no weight loss was observed for the SH1-3 μg prime-only group. Mice in the prime-only vaccination groups that received lower vaccine doses (0.3 μg and 0.03 μg) showed more weight loss (7% and 10%, respectively) than mice in the high dose or prime-boost groups (both 3%), but the mice were completely protected from mortality and regained weight after day 5 post challenge (Fig. 1A and C). Mice vaccinated with AH1-VLPs lost slightly more weight than mice that received the same dose of SH1-VLPs (0.3 μg of HA) but were fully protected from mortality (Fig. 1A and C). Animals that received an M1-only preparation containing similar amounts of baculovirus as the SH1- and AH1-VLP preparation showed no enhanced protection as compared to naïve mice (Fig. 1A and C). This proves that neither M1 or the baculovirus or a combination of both was able to induce significant protective immune responses in our challenge model. Since previous studies highlight the critical role of CD8^+^ T-cells in protective immunity to influenza infection [26], [27], we assessed whether a single low vaccine dose could also induce full protection in CD8^+^ T-cell-depleted mice. Minimal weight loss for CD8^+^-depleted, SH1-0.3 μg-vaccinated mice after challenge and a 100% survival rate (Fig. 1B and D) suggested that the humoral response was sufficient to robustly protect these animals.

### 3.2 H7 VLP vaccines induce broadly reactive antibodies

**Fig. 2:**
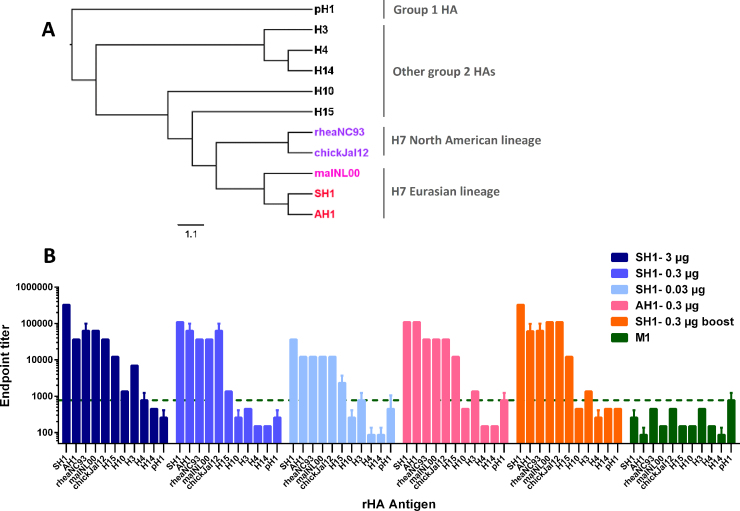
VLP vaccines induce broadly reactive antibodies in mice. Sera from vaccinated mice were assayed for antibody endpoint titres against representatives of all group 2 HAs and one H1 HA (group 1). (A) Phylogenetic analysis was performed with the two vaccine strains (red), another representative of the H7 Eurasian lineage (pink), more divergent HAs from the North American lineage (purple) and other HA representatives of group 2 and group 1 (both black) that were evaluated in the ELISA. Phylogenetic trees were constructed using the CLUSTAL W algorithm using the neighbour-joining method and visualised with FigTree. (B) Samples were from mice (*N* = 5) immunised with a single dose of SH1-VLPs containing 3 (dark blue), 0.3 (signal blue) or 0.03 μg HA (light blue), AH1-VLPs (pink) with a HA-content of 0.3 μg, two doses of SH1-VLPs (orange; days 0 and 14) with a HA-content of 0.3 μg or M1-VLPs (dark green) administered at a total protein concentration equal to the SH1-0.3 μg vaccine dose. Mice were bled 35 days post prime or 21 post boost respectively and assayed for the indicated panel of rHAs. Pooled serum was used to assess reactivity to the eleven different HAs. Values represent the geometric mean of the calculated end point titres (read-outs subtracted by the mean and three-fold standard deviation) and error bars depict the standard error of the mean. The green baseline indicates background levels of serum from mice that received the M1-VLP (no HA included).

As previous studies reported a remarkable cross-reactivity of H7 antibodies [13], [28], we tested sero-reactivity to a panel of divergent recombinant H7 proteins and a representative HA from each influenza subtype (H3, H4, H10, H14 and H15 – in addition to H7) that cluster into phylogenetic group 2 (Fig. 2A). An H1 HA (group 1) was added as a negative control antigen. Strong sero-reactivity was detected against the HA of the vaccine strains SH1 and AH1. Other representative strains of the Eurasian lineage (malNL00) and more divergent H7 strains from the North American lineage (rheaNC99, chickJal12) showed endpoint titres only up to 3-fold lower than were achieved with the HA of the vaccine strain (Fig. 2B). Good correlation could be drawn between vaccine dose and total IgG levels to homologous and heterologous H7 strains as seen by the dose-dependent decrease of antibody levels in most cases. Moreover, we could detect considerable cross-reactivity against subtypes H15 and H3 across all tested sera.

### 3.3 H7 VLPs induce cross-reactive HI active antibody responses

Levels of neutralising antibodies elicited by each vaccine were measured by hemagglutination inhibition (HI) assay in which sera from vaccinated mice were evaluated for their ability to prevent virus-induced agglutination of turkey RBCs. Results show that the VLP-based H7 vaccine induced high HI-active antibody titres up to 1:40 for PR8:SH1 and up to 1:80 against PR8:AH1 (Table 1). Both VLP vaccines were also able to induce levels of HI antibodies that prevented virus-induced hemagglutination by a panel of divergent H7 strains of the Eurasian and the more distant North American lineage, with titres of up to 1:40. Single vaccination with the two higher SH1-VLP vaccine doses (3 μg and 0.3 μg) generated similar amounts of HI-active antibodies for all tested virus strains and negligible HI titres were measured for the lowest vaccine dose (0.03 μg). The second dose of SH1-VLP vaccine led to a 2-fold enhancement of average levels of HI-active antibodies for most of the virus strains tested. No HI antibodies were detected in the two control groups (naïve and M1-vaccinated mice).

**Table 1:** HI antibody titres induced by vaccination with various H7-VLP vaccine doses.

Vaccine group^a^	HI titre (1:*X*) against recombinant virus strains^d^				
	PR8:SH1^b^	PR8:AH1^c^	PR8:malNL00^c^	PR8:malAlb01^c^	PR8:chickJal12^c^
Naïve	<10.0 (all <10)	<10.0	<10.0	<10.0	<10.0
SH1-3 μg	15.2 (10–40)	40.0	10.0	20.0	10.0
SH1-0.3 μg	15.2 (10–20)	40.0	10.0	20.0	10.0
SH1-0.03 μg	5.7 (<10–10)	10.0	<10.0	7.1 (<10–10)	<10.0
AH1-0.3 μg	10.0 (<10–20)	20.0	10.0	20.0	7.1 (<10–10)
SH1-0.3 μg boost	20.0 (10–40)	80.0	20.0	28.3 (20–40)	10.0
M1	<10.0	<10.0	<10.0	<10.0	<10.0

a Mice (*N* = 5 per group) were immunised once intramuscularly with SH1-VLPs or AH-1VLPs at indicated concentrations based on the HA content. One group (SH1-0.3 μg boost) received two vaccine doses with the indicated HA content. Control groups were naive mice or mice vaccinated once with M1-VLPs at a total protein concentration equal to the SH1-0.3 μg-VLPs vaccine dose.

b Sera from individual mice were assayed for A/Shanghai/1/13 (SH1). Shown are the geometric mean and the range (in brackets).

c HI titres for divergent H7 strains were assayed with pooled sera in duplicates.

d HI titres are expressed as the reciprocal of the highest dilution of serum that completely inhibited agglutination of turkey red blood cells by four HA units of the five titrated viruses (PR8:SH1, PR8:AH1, PR8:malNL00, PR8:malAlb01 and PR8:chickJal12). HI titres are indicated as geometric mean values. In case the duplicate measurements were both negative, the HI titre was assigned as <10. Single negative results in a duplicate measurement were assigned a value of 5 for the calculation of the geometric mean value. The range of the duplicate measurements is given in brackets if it yielded two different values.

## 4 Discussion

On 31 March 2013, the Chinese Health and Family Planning Commission notified the WHO of three cases of human infections with a novel influenza A (H7N9) strain [1], which has been the causative agent for 137 infections with a mortality rate of 33% as of 25 October. It remains unclear whether the virus will persist in the human reservoir and cause sporadic infections, or whether it will gain the ability to transmit from human to human through mutations or re-assortment [29]. Limited reports on new human incidences might be due to the shutdown of live poultry markets throughout the country. However, H7N9 may also follow a seasonal outbreak pattern similar to H5N1, therefore an epidemic could re-occur in fall [30]. Since no vaccine for H7N9 is available for humans, antivirals are the only treatment options, but bear the risk to yield treatment-resistant viruses, which were already associated with poor clinical outcome [6], [7]. The potential threat of a pandemic outbreak serves as catalyst for enhanced research and vaccine development efforts in both academia and industry. Human H7 vaccines are underway and have been evaluated in preclinical [8], [9], [10], [11], [13], [14], [31], [32] or phase I or I/II studies [12], [33], [34], [35]. However, most H7 vaccines showed low immunogenicity in humans and might not be able to raise titres of HI-active antibodies in the protective range [34], [35]. Furthermore, the overall majority of H7 vaccines in the pipeline are focused on egg-based production which might be an inadequate platform in a pandemic setting due to limited manufacturing capacities and longer production times compared to cell-culture based systems. Based on predictions that consider the current maximum global capacity for influenza virus vaccine manufacturing vaccine production will be too slow to adequately meet the needs for a vaccine in the event of a pandemic [36]. A major factor limiting the manufacturing capacity of a vaccine is the minimum immunogenic antigen dose that confers protection. It is highly desirable to obtain good efficacy already with low vaccine doses and the fewest possible injections to prevent shortages. Development of more efficient vaccines is a key objective defined by the Global Action Plan for Influenza vaccines by the WHO [37].

Here, we chose to evaluate a low-dose single-shot VLP vaccine against the novel H7N9 virus. Single immunisation with as low as 0.03 μg SH1-VLP preparation (based on HA content) could confer full protection against a stringent homologous challenge (100 mLD50) in BALB/c mice (Fig. 1C). Mice that were vaccinated with a single vaccine dose of 3 μg SH1-VLP did not show any sign of disease. This is in contrast to an earlier study by Smith et al. who reported that mice vaccinated with a two dose regimen with 0.7–2 μg lost 10–15% of their initial body weight after a 3.5 LD50 challenge [14]. Since the VLPs used in their study were highly purified we would speculate that active baculovirus contaminants in our vaccine preparations (supplementary data) acted as an adjuvant and boosted the immune response – an effect that was reported before. It was shown that baculovirus can enhance immunogenicity of VLP vaccines through boosting the immune response by interferon-signalling and biasing IgG isotype distribution [16]. Vaccination with VLPs harbouring an HA from a closely related (but phylogenetically distinct) H7 strain, A/Anhui/1/13, also protected mice from PR8:SH1 challenge after only one immunisation.

Generally, T-lymphocytes have long been appreciated as a critical contributor to protection and recovery from influenza infection [38]. Essentially, CD8^+^ T-cells play an important role in the clearance of virus infected cells and thereby limit viral replication, disease development and reduce mortality [26], [38], [39]. We tended to address the importance of the cytotoxic immune response mediated by CD8^+^-cells in our challenge experiment. CD8^+^-depleted mice were fully protected in the challenge experiment and showed similar weight loss kinetics as observed for non-depleted mice (Fig. 1B and D), which is in agreement with previous findings [40]. However, in a recent work by Hemann et al. [26], only 60% survival of CD8^+^-depleted mice that received a single dose of an influenza H1-VLP vaccine was reported in a homologous challenge experiment after intranasal vaccination with 2.5 μg VLPs. This suggests that our VLP preparation induces sufficiently high titres of neutralising antibodies, even at low single vaccine doses of 0.03–0.3 μg VLP, to be protective in a stringent homologous and heterologous challenge. A contribution of virus-specific CD8^+^-cells to protection from infection might be redundant in this case. As the delivery route of VLPs was shown to influence the strengths of the humoral and cellular immune response [16], [41], one might speculate whether the survival rate would have been higher in the study of Hemann et al. [26], if an alternative to the intranasal vaccination route was chosen.

Single immunisations with our vaccine could induce antibodies that were reactive to all heterologous H7 subtypes tested (Fig. 2), in agreement with an earlier study [13]. We could also demonstrate significant reactivity to other members of group 2 HAs, such as the phylogenetically related H15 subtype and the more divergent H3 HA. Interestingly, cross-reactivity to H10, which is phylogenetically closer to H7 than H3, was only slightly above the background signal for the 3 μg dose group (Fig. 2), which is in agreement with results recently obtained by Muramatsu and colleagues [42]. It was previously shown that vaccination with different immunogens that vary only in their globular head region, specifically could boost the stalk-reactive antibody response in mice [22], [43]. However, both our immunisations for the prime-boost group were performed with the same immunogen and we assume that the boost in sero-reactivity primarily results from head-specific antibodies. We therefore investigated the activity of the elicited antibodies by a hemagglutination inhibition assay with a panel of H7 strains. HI-active antibodies could be detected for the vaccine strains but also for a panel of divergent H7 viruses, which included representatives of the Eurasian and the North American lineage (Table 1). These results are in good agreement with those from Abbas et al. [44] obtained in chicken and Goff et al. [13] and Smith et al. [14] obtained in mice. We detected lower HI-activity for the PR8:SH1 virus than for PR8:AH1, even for the groups immunised with SH1-VLPs. This may be due to the utilisation of individual *versus* pooled sera in the assays. Although virus preparations were standardised, there still might have been slight variations in HA-activity of the viruses utilised. The second immunisation leads to a two-fold increase in HI titres for almost all tested virus strains. The observed HI crossreactivity might be the result of the completely conserved antigenic site A of Eurasian and North American lineage H7 viruses [13]. It is of note that even the group that received the lowest VLP dose of 0.03 μg and had only neglectable HI-activity was completely protected from challenge, suggesting that detectable levels of HI-active antibodies might not be required for protection.

## 5 Conclusion

Here we show full protection of mice vaccinated with low doses of an H7-VLP vaccine based on the HA antigens of the novel H7N9 strains in a one-shot vaccination strategy from a stringent lethal challenge with an H7N9 virus. Furthermore, these VLPs induced broad sero- and HI-reactivity. Based on this data we speculate that the vaccine could also protect against other, divergent H7 strains. We have previously shown that the presence of active baculovirus in insect cell-derived VLP preparation is able to substantially increase immunogenicity and protection due to its immune-stimulatory capability [16]. We would assume that they play a substantial role in the efficacy and potent immunogenicity of the H7 VLP vaccine tested here. VLP vaccines that contain baculoviruses might prove to be useful in pandemic situations where large quantities of highly effective vaccines are needed. However, bioactive, live viruses in vaccine formulations might induce strong reactogenicity and safety concerns might prevent their application in humans. Importantly, a bioactive baculovirus component of a vaccine would need to be standardised and tested for stability under different storage conditions. In addition it would be necessary to assess the minimum effective concentration of baculovirus in a vaccine dose and to establish an acceptable HA or VLP to active baculovirus ratio. Assessment of the latter ratio might be difficult due to the presence of baculovirus–VLP hybrids – baculovirus particles that incorporate HA and VLPs that incorporate baculovirus capsid and envelope proteins [45], [46]. As a large body of research is currently focusing on baculovirus-based expression systems in vaccine manufacturing, more safety data will accumulate and more analytical methods will become available for this system in the near future [46], [47] and might possibly spur its establishment in human applications.
