# Supplementary material for: One-shot vaccination with an insect cell-derived low-dose influenza A H7 virus-like particle preparation protects mice against H7N9 challenge
Source: Vaccine. 2014 Jan 9;32(3):355–62. doi: 10.1016/j.vaccine.2013.11.036 (PMC3906608; doi:10.1016/j.vaccine.2013.11.036)

| **Vaccine^a^** | **HA content**  **(µg)^b^** | **Baculovirus titer**  **(pfu/mL)^c^** | **Infectious baculovirus per amount of HA**  **(pfu/µg)** | **Infectious baculovirus per vaccine dose**  **(pfu/dose)** |
| --- | --- | --- | --- | --- |
| **SH1-3µg** | 3 | 1.10 x 10^9^ (±0.54) | 1.83 x 10^7^ | 5.50 x 10^7^ |
| **SH1-0.3µg** | 0.3 | 1.10 x 10^8^ (±0.34) | 1.83 x 10^7^ | 5.50 x 10^6^ |
| **SH1 -0.03µg** | 0.03 | 1.57 x 10^7^ (±0.67) | 2.62 x 10^7^ | 7.85 x 10^5^ |
| **AH1-0.3µg** | 0.3 | 0.62 x 10^8^ (±0.19) | 1.03 x 10^7^ | 3.10 x 10^6^ |
| **M1** | - | 0.25 x 10^8^ (±0.14) | NA | 1.25 x 10^6^ |

**Supplementary Table 1: Baculovirus content in the vaccine preparations**

^a^ Nomenclature of the different vaccine preparations used for vaccination of mice.

^b^The HA content of the different preparations was determined by densitometrically as described in Material and Methods and is indicated for each vaccine dose. M1-VLPs do not contain HA and were administered at a total protein concentration equal to that of one SH1-0.3µg dose.

^c^Baculovirus background titers were measured by plaque assay as described in Material and Methods. Titers represent the arithmetic mean of triplicates and values in brackets indicate the standard deviation.

**Supplementary Method: Sucrose-gradient centrifugation**

Co-migration of the VLP components of the vaccine preparations was evaluated by sucrose-gradient density centrifugation. 0.2 mL SH1-VLPs and M1-VLPs that were pelleted and resuspended in PBS were loaded on a 20 – 60% (w/v) sucrose gradient in NTE buffer (100 mM NaCl, 10 mM Tris-HCl pH 7.4, 1 mM EDTA) to yield a total sample volume of 5 mL. VLPs were centrifuged for 16 hours, 4°C at 190.000 g with a SW55Ti rotor (Beckman Coulter, Brea, CA). After centrifugation, the gradient was split up in 12 fractions and the pellet fraction and examined on a Western blot as described before. Briefly, the membranes were incubated for 1 h with reconvalescent serum obtained from mice sublethaly infected with H7N1 at a dilution of 1:1000 and with mAb E10 at a dilution of 1:2000 in PBS containing 0.1% Tween 20 (v/v) (PBST) and 1% (w/v) non-fat dry milk powder. Membranes were further processed for chemiluminscence detection of VLP components as described before.

**Supplementary Figure 1:** **VLP Sucrose gradient centrifugation**

Twelve fractions and the pellet fractions of the gradient centrifugation were visualised on a Western blot by chemiluminscent detection using reconvalescent mouse serum from H7N1 infected animals as probe. **(A)** SH1-VLP fractions and **(B)** M1-VLP fractions 1-12 are loaded from left to right with increasing sucrose density, terminating with the pellet fraction (P).


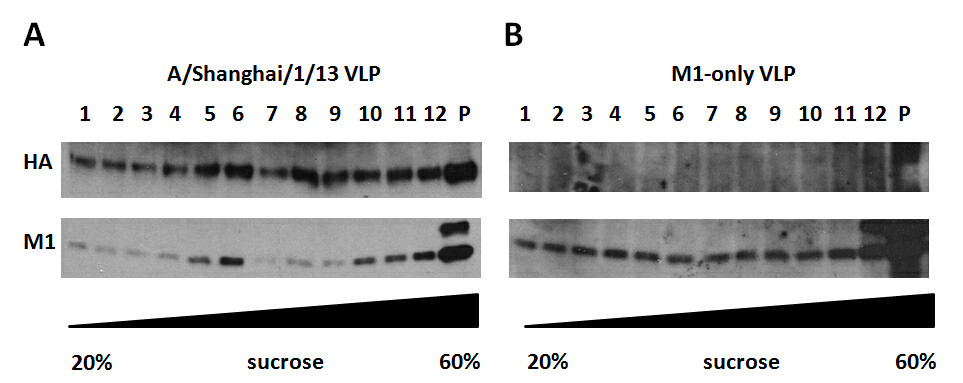

Supplement: Supplementary file 1 [file mmc1.docx]
